# Gene overexpression screen for chromosome instability in yeast primarily identifies cell cycle progression genes

**DOI:** 10.1007/s00294-018-0885-x

**Published:** 2018-09-22

**Authors:** Hanna Tutaj, Elzbieta Pogoda, Katarzyna Tomala, Ryszard Korona

**Affiliations:** Institute of Environmental Sciences, Jagiellonian University, Gronostajowa 7, 30-387 Kraków, Poland

**Keywords:** *Saccharomyces cerevisiae*, Chromosome loss, Chromosome recombination, 2-Micron plasmid, Mitosis

## Abstract

Loss of heterozygosity (LOH) in a vegetatively growing diploid cell signals irregularity of mitosis. Therefore, assays of LOH serve to discover pathways critical for proper replication and segregation of chromosomes. We screened for enhanced LOH in a whole-genome collection of diploid yeast strains in which a single gene was strongly overexpressed. We found 39 overexpression strains with substantially increased LOH caused either by recombination or by chromosome instability. Most of them, 32 in total, belonged to the category of “cell division”, a broadly defined biological process. Of those, only one, *TOP3*, coded for an enzyme that uses DNA as a substrate. The rest related to establishment and maintenance of cell polarity, chromosome segregation, and cell cycle checkpoints. Former studies, in which gene deletions were used, showed that an absence of a protein participating in the DNA processing machinery is a potent stimulator of genome instability. As our results suggest, overexpression of such proteins is not comparably damaging as the absence of them. It may mean that the harmful effect of overexpression is more likely to occur in more complex and multistage processes, such as chromosome segregation. We also report a side finding, resulting from the fact that we worked with the yeast strains bearing a 2-micron plasmid. We noted that intense transcription from such a plasmid led to an enhanced rate of an entire chromosome loss (as opposed to LOH produced by recombination). This observation may support models linking segregation of 2-micron plasmids to segregation of chromosomes.

**Electronic supplementary material:**

The online version of this article (10.1007/s00294-018-0885-x) contains supplementary material, which is available to authorized users.

## Introduction

Events of unequal distribution of DNA to daughter cells upon mitosis can be collectively described as chromosomal instability (CIN). One approach to study CIN is to detect and compare the existing aberrant genomes. However, chromosomal mutations are typically non-neutral to fitness. Some can be beneficial and fixed by natural selection (Comai 2005; Harari et al. 2018; Otto and Whitton 2000; Torres et al. 2008). More frequently, they are deleterious and, therefore, eliminated either immediately or quickly enough to pass overlooked (Sheltzer and Amon 2011). The deleterious changes known from genetic counselling constitute probably only a small and non-random sample of all such events (Duijf and Benezra 2013; Hui and Bianchi 2013). It is, therefore, advisable to do systematic genetic screens of possibly nascent CINs, even though they usually involve specifically engineered and thus rather narrowly focused detection systems (Duffy et al. 2016; Dunham and Fowler 2013).

Genome-wide screens for factors affecting a trait of question are routinely done with a handful of model species of which *Saccharomyces cerevisiae* is outstanding both in terms of opportunities offered and results already obtained (Boone 2014; Giaever and Nislow 2014; Scherens and Goffeau 2004). The previous systematic screens for genes affecting CIN in this species can be divided according to two criteria. One is the way in which activity of genes potentially linked to CIN was affected: either completely absent (deletion studies) or just modified (expression studies). The other is the kind of sensor of genetic change: either loss of an additional chromosome fragment (within a haploid or diploid cell) or loss of heterozygosity (LOH) at a locus residing on a regular chromosome of a diploid cell. Combining these two criteria results in four possibilities. The combination of an artificial detector and alteration of gene expression have been tried several times: with strong overexpression from the *GAL1* promoter (Duffy et al. 2016; Ouspenski et al. 1999), mild underexpression from hemizygous loci, and mild overexpression conferred by centromeric plasmids with genes under own promoters (Zhu et al. 2015). Two other combinations involved deletion strains (complete loss of gene function) and either a chromosomal fragment (Yuen et al. 2007) or intact natural chromosomes (Andersen et al. 2008; Choy et al. 2013; Kanellis et al. 2007; Yuen et al. 2007). The lists of genes identified in particular studies tended to overlap substantially and all were clearly enriched in functional categories known to be important for the stability of genomes. On the other hand, each of those studies pointed to a number of new genes as factors affecting genetic stability in yeast. One reason why results differ between screens is that studies relying on additional fragments of chromosomes tend to perform well in detecting chromosome loss, while those relying on natural chromosomes are better suited to reveal recombination (Acuña et al. 1994; Andersen et al. 2008; Yuen et al. 2007). Another is that an absence of a protein is likely to disturb the cell functioning in a different way than its excess (Oberdorf and Kortemme 2009; Veitia et al. 2013). In sum, the already used approaches to screening for the CIN genes provided both new hits and reassuring cross validations. However, it is still likely that a non-trivial number of potentially important genes has not been yet identified (Duffy et al. 2016; Stirling et al. 2011).

We set to expand systematic studies of CIN by introducing the lacking fourth combination: overexpression of single genes with LOH at natural chromosomes as detectors of instability. In our case, overexpression was very strong, because we used multi-copy plasmids with genes fused to the *GAL1* promoter. This could result in intensification of regular functions of genes, but not only. Possible outcomes include excessive sequestration of partners, competition for subunits shared with other proteins, disruption of stoichiometric complexes, promiscuous interactions with proteins or nucleic acids, toxicity of misfolded aggregates, and others (Albrecht et al. 2018; Moriya 2015; Prelich 2012). Thus, overexpression studies can lead to unexpected and interesting results by creating perturbations which are rather artificial but, nevertheless, revealing.

We performed a genetic screen for increased rate of LOH in *S. cerevisiae*. We used a complete genomic collection of 2 µ plasmids each carrying a single yeast gene under strong inducible promoter (Gelperin et al. 2005). LOH was marked by mutation to canavanine resistance which occurred when a functional allele was lost at a heterozygous *CAN1*/*can1* locus residing on the chromosome V of the host cell (mutations inactivating *CAN1* are much rarer). A co-occurring LOH at *MET6*/*met6* on the opposite arm of the same chromosome signaled that an entire chromosome V was lost. The canavanine-resistance assay was quantitative, it allowed to estimate how much the frequency of LOH increased, and how often, it was caused by the loss of an entire chromosome. We provide a list of genes which caused a substantial, and often dramatic, increase in LOH upon strong overexpression. We also show that chromosomal instability depended on the identity and not amount of overexpressed protein even if the latter was enhanced by orders of magnitude.

## Materials and methods

### Strains and plasmids

We used a previously constructed collection of single yeast open-reading frames (ORFs), each with an inducible promoter P_*GAL1*_, cloned into a multi-copy 2-micron plasmid with the *URA3* marker and hosted by the haploid yeast strain Y258 *MAT***a***pep4-3 his4-580 ura3-52 leu2-3*. This so-called MORF collection is used to express proteins with a C-terminus fused affinity tag His_6_-HA-ZZ. As much as 93% of 5188 such constructs could be recovered from cell lysates and most of those appeared also to be intact in vivo (Gelperin et al. 2005). The host stain is derived from the S288c background (http://dharmacon.gelifesciences.com/resources/faqs/y258-used-yeast-orf-collection-derived-s288c).

**Fig. 1:**
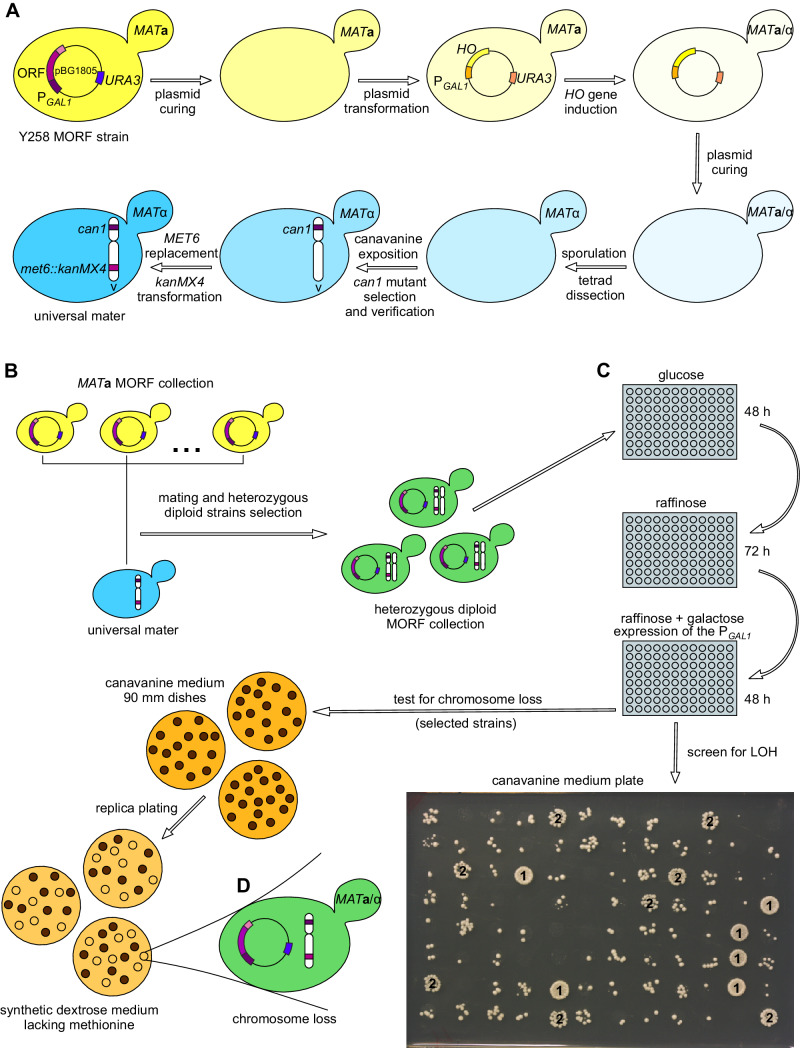
Overview of the experiment. **a** Derivation of a Y258 *MATα* strain. The final construct was strictly isogenic to the original *MAT***a** strain (host of the whole collection) except for the presence of two markers introduced to chromosome V and absence of a MORF plasmid. **b***MATα* strain was then used as a universal mater to create a collection of heterozygous strains bearing MORF plasmids with single yeast genes under an inducible promoter, P_*GAL1*_. **c** Test for enhanced LOH. Cultures were gradually induced to high expression of genes residing on the MORF plasmid. Aliquots of 5 µl were exposed to canavanine medium yielding highly variable numbers of Can^R^ colonies. A group of 636 candidate strains selected after an initial whole-genome screen was tested in two additional trials and ranked as very dense (1), dense (2) and standard (no labels). Those which exhibited consistently enhanced LOH in further tests (see the main text) were assayed for the frequency of chromosome V loss which was marked by an inability to grow on media-lacking methionine (**d**)

Figure 1 summarizes methods used to derive and assay our experimental strains. As the intention of the study was to test for stability of chromosomes in a mitotically dividing diploid cell, we had to turn the entire MORF collection of haploids into diploids. We first cured one of the haploid strains of its MORF plasmid and transformed it with another plasmid, p*URA3* P_*GAL1*_-*HO*. After transient induction of the *HO* gene with galactose, a diploid strain was obtained, cured of the plasmid, sporulated, and dissected to derive an *MATα* haploid strain. This new strain was a stable haploid and fully isogenic to the original Y258, except for the mating type. Two further changes were introduced into it. First, we exposed it to 60 mg/l canavanine to select for Can^R^ mutants. One of them, confirmed to have a frame-shift mutation in *CAN1*, was selected for the next step, that is, replacing the *MET6* gene with a cassette providing resistance to geneticin, yielding *met6::kanMX4*. (*CAN1* and *MET6* reside, respectively, on the left and right arms of chromosome V.)

The obtained *MATα* strain was used as a single mating partner for all *MAT***a** plasmid-hosting strains. The mating was done by mixing aliquots of 10 µl of the *MATα* strain with the same volume of a single MORF strain in 96-well titration plates and adding 180 µl of fresh YPD. Diploids were selected through three consecutive transfers into synthetic minimal glucose (SD) medium supplemented with leucine, histidine, and 200 mg/l geneticin. The resultant collection of the overexpression plasmids in double heterozygous diploid cells, *CAN1*/*can1 HIS6*/*his6::kanMX4*, was stored at –70 °C. To keep the P_*GAL1*_ promoter repressed, the only carbon source used when handling both the haploid and diploid collections was glucose.

### Screen for LOH with canavanine-resistance assay

Frozen samples of the diploid collection were thawed and transferred with a 96-pin replicator onto SD agar supplemented with leucine, histidine, and geneticin. In this and all other experiments, the temperature of incubation was kept at 30 °C. The P_*GAL1*_ promoter is turned on in two steps. First, its repressor (glucose) is removed, and this is done using raffinose as carbon source. Then, galactose acting as an activator is added (Gelperin et al. 2005). In our experiment, after initial micro-cultures reached the stationary phase density in glucose, samples of 2 µl were transferred to 198 µl of the same glucose-based medium. The second transfer to glucose was done to synchronize the growth phase of the micro-cultures. After 48 h of incubation, 20 µl samples were transferred to 180 µl of synthetic complete (SC) medium lacking uracil and with 2% raffinose replacing glucose. After 72 h of incubation, 5 µl samples were transferred to 195 µl of new medium, the same as above plus 2% galactose (Raffinose was required as Y258 does not utilize galactose as a source of energy). After 48 h of incubation, 4 µl samples were put onto agar SC medium (96 per one rectangular plate) which had 2% glucose as the sole source of carbon, lacked arginine and was supplemented with 60 mg/l canavanine. The agar plates were photographed after 48 h and then kept at 4 °C. Enlarged photographs and the original plates were evaluated independently by two experimenters looking for visible enrichment in the number of Can^R^ mutants (identity of the evaluated strains was unknown during this inspection). The criteria used to decide about enhanced LOH are described in “Results”.

### Test for chromosome loss with methionine auxotrophy assay

This experiment was done in the same way as the LOH screen except for the final transfer of samples of galactose-induced cultures onto agar with canavanine. These samples were placed not as 96-spot arrays but on individual 90 mm dishes. The volume of individual samples varied, and the goal was to obtain about 200 colonies of Can^R^ mutants per plate which then could be replica plated onto SD agar supplemented with leucine and histidine but not methionine. At least three plates per every strain classified as one with enhanced LOH were replica plated.

### Other methods

To measure the level of protein overexpression, the studied strains were transferred through media based on glucose, raffinose, and raffinose with galactose in the way described above. In the last medium, cells were harvested after 24 h of incubation, washed with ice cold water and frozen. To start protein extraction, the cells were beaten with glass beads in 100 µl of lysis buffer (50 mM Tris–HCl, pH 7.5, 0.5% SDS, 0.1 mM EDTA, protease inhibitors) for 4 h in 4 °C. Afterwards, cell remnants were spun down and supernatant was collected. Total protein content was determined using a BCA protocol. The same supernatant was used for a competitive ELISA assay aimed at determining the cellular level of the overexpressed proteins (Tomala and Korona 2013).

The cellular content of reactive oxygen species was determined with CellROX^®^ Deep Red dye (Life Technologies). Cells were grown and harvested in the same way as in the protein assay. The dye was added at the working concentration of 5 µM. After the dying, samples of cells were washed in PBS and lysed with lithium acetate and SDS. After spinning down the cell debris, supernatants of the resulting samples were measured for fluorescence with 635 nm of the excitation and 665 nm of emission wavelength. The use of cell extracts, instead of intact cells, has been found more reliable for this particular dying kit (James et al. 2015).

## Results

### Can^R^ screen for increased LOH

We screened a set of 5179 diploid strains carrying single overexpression plasmids, arrayed individually on 96-well titration plates. We grew them on medium containing galactose to induce overexpression and then transferred samples onto agar plates with canavanine (and glucose to stop overexpression). The size of samples was adjusted to yield 5–10 Can^R^ colonies as the most frequent result. We found 636 candidates for enhanced LOH by applying an intentionally liberal criterion: the number of colonies had to be at least three times higher. Supplementary Table S1 lists these genes. This set was likely to include a fair number of strains with “standard” rate of LOH but ones in which an enhanced number of mutants occurred by chance. To narrow the search, all 636 candidate strains were re-assayed in two independent runs. This time, densities of Can^R^ colonies per patch were classified into three ranks of increasing LOH. Figure 1 illustrates how the patches of colonies were divided into three ranks: standard, dense, and very dense, that is, filled with colonies. Ranks of the two replicas generally did correlate with each other (Pearson’s *r* = 0.501; *t* = 14.576, *df* = 634, *p* << 0.0001). However, two replicas of a single strains often differed considerably. It was understandable given the random nature of mutational events, but it also undermined reliability of any decision about the impact of individual genes on LOH. Therefore, we grouped the 636 strains into GO (Gene Ontology) functional categories. We then calculated a mean rank of patch density for every category (for those with more than 4 members among the 636 candidates). The category ranks were compared with the mean rank of a control group of 200 strains (randomly chosen from among the non-candidate strains). We found a statistically significant difference for 11 categories (partially overlapping) with 97 unique genes (Supplementary Table S2).

### Genes enhancing LOH

**Fig. 2:**
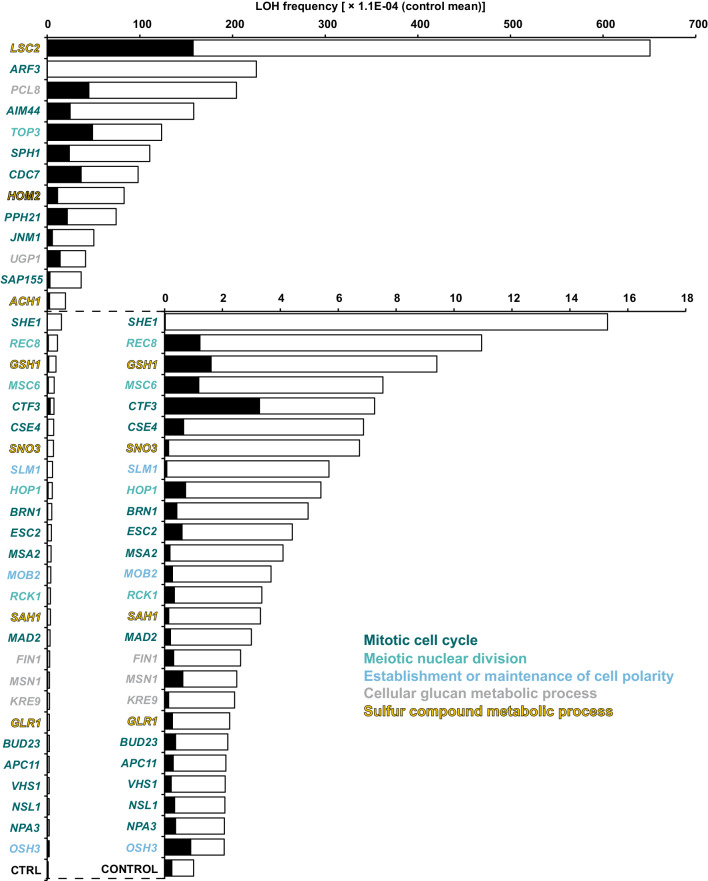
Genes which increase frequency of LOH more than twice when overexpressed. Frequency of LOH at the *CAN1*/*can1* locus is represented by filled bars. LOH at both *CAN1*/*can1* and *MET6*/*met6* (loss of chromosome V) is represented by empty bars. Control consisted of 28 strains randomly selected from among those in which LOH was observed with about median frequency (see the main text). Average frequency of total LOH in the control 28 strains was 1.01 × 10^− 4^ and is used as a unit to scale the horizontal axis

The 97 genes were subjected to further tests to confirm that they indeed increased LOH and find out how often LOH resulted from the loss of an entire chromosome. For each tested strain, replicate samples of size sufficiently large to contain hundreds of Can^R^ mutants were used. After the Can^R^ colonies were counted, they were replica plated to test for the Met^−^ phenotype marking LOH at the *MET6* locus. Figure 2 shows 39 (out of 97) strains in which the mean frequency of LOH at the *CAN1* locus was confirmed to be higher (at least two times) than in the control strains (control was composed of 28 randomly chosen strains which showed about median LOH in former tests). Both in the control and the confirmed 39 strains, LOH at *CAN1* was frequently accompanied by LOH at *MET6*. We assumed that a double LOH indicated the loss of an entire chromosome V. Not only because double recombination, double mutation or simultaneous recombination at one marker and mutation at the other are less likely to occur than a loss of a chromosome. We also observed that the Can^R^ Met^−^ colonies were distinctly smaller than the Can^R^ Met^+^ ones indicating growth disadvantage typical for monosomics (Alvaro et al. 2006).

Regarding functions of the 39 genes, 32 of them relate to cell division. Most of them are known to be needed at different stages of mitosis beginning with establishment of cell polarity until the late cell division checkpoints. Only few—*CDC7, HOP1, MSC6, RCK1*, and *REC8*—are thought to participate in meiosis, and therefore, it might have been destructive to express them under vegetative growth.

**Fig. 3:**
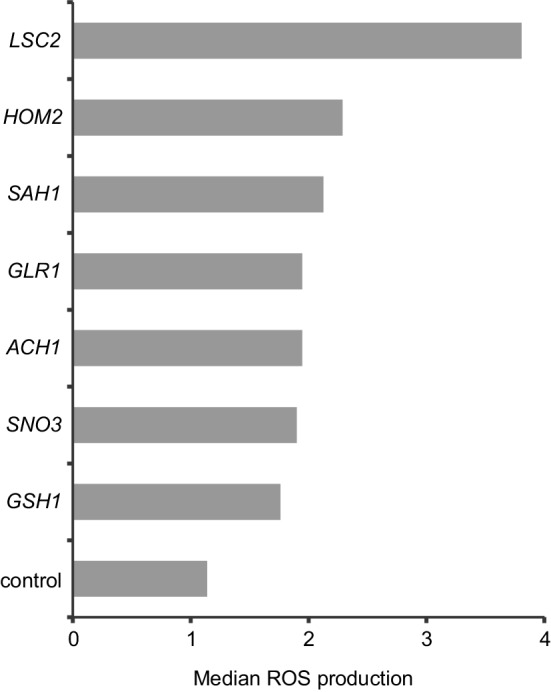
Cellular level of ROS after overexpression. The seven genes belonging to the category of metabolism of sulfur compounds are compared with a control obtained as a mean of three randomly chosen genes judged “standard” (see Fig. 1)

The seven remaining genes were categorized by a Gene Ontology analysis tool as involved in “metabolism of sulfur compounds” (Eden et al. 2009b). They have no clear links to cell division, but could possibly destabilize functioning of the cell, including cell division, if overabundance of their products resulted in an excessive level of reactive oxygen species (ROS). *LSC2*, which upon overexpression destabilized the genome more than any other gene, appears to also excite ROS to the highest level (Fig. 3).

### Frequent loss of an entire chromosome in the presence of galactose

The median frequency of Can^R^ mutants in the control was 1.1 × 10^− 4^ which is about average for LOH in a mitotically dividing diploid yeast cell (Andersen et al. 2008). More intriguing was the observed high frequency of double LOH, because in *S. cerevisiae* the combined frequency of events involving chromosome recombination, breakage, or repair is typically higher than the loss of an entire chromosome (Andersen et al. 2008; McMurray and Gottschling 2003).

**Fig. 4:**
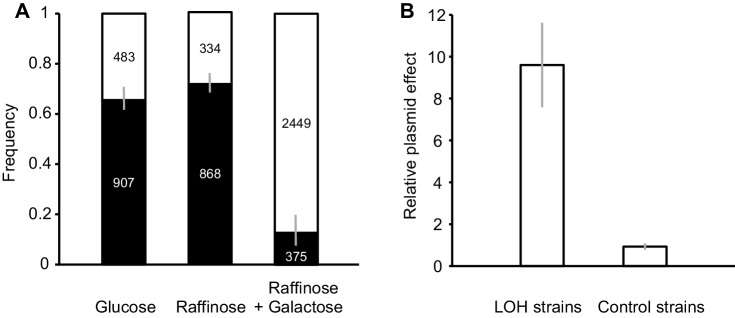
**a** Relative frequencies of the two types of LOH in the control strains: single LOH at the *CAN1*/*can1* locus (black field) and double LOH at both the *CAN1*/*can1* and *MET6*/*met6* loci (open field) with SE shown as bars. Numbers of colonies used to calculate the frequencies are shown in relevant fields. Cultures were passed sequentially through the three media used to induce overexpression—SC with the source of organic carbon being glucose, raffinose or raffinose with galactose—with LOH being scored at the end of each phase of growth. **b** Effect of a plasmid on the frequency of LOH. The tested 39 strains and control 28 strains (compare Fig. 2) were cured of plasmid. The ratio of the frequency of LOH with and without plasmid was calculated for each strain and averaged for the two groups. Bars show standard errors

We hypothesized that the observed high frequency of the loss of chromosome could depend on the specific medium we used. We repeated the tests of LOH for the control strains with the medium containing raffinose and galactose but also with media containing only glucose or only raffinose. Figure 4a shows that simultaneous LOH at both loci was less frequent than single LOH at *CAN1* when glucose or raffinose was used. Thus, the strain used by us was not specific, or defective, in a way that would make the loss of an entire chromosome especially frequent. Only for raffinose plus galactose, when the P_*GAL1*_ promoter was fully active, the loss of an entire chromosome prevailed over other events underpinning LOH. Figure 4b demonstrates that galactose is a stimulator of LOH only when an MORF plasmid is present. This result provides no support for an alternative hypothesis stating that galactose may be a metabolic agent disturbing the cell division regardless of its interaction with the target promoter.

### Rate of LOH was determined by the function, not amount of overexpressed protein

**Fig. 5:**
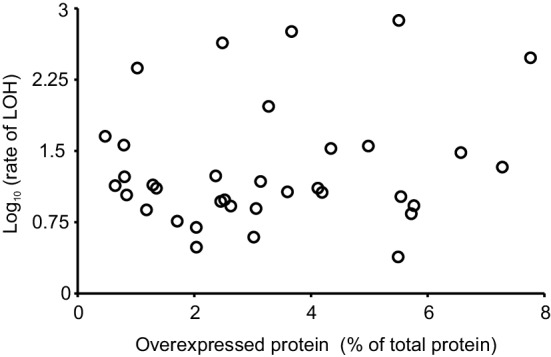
Relation between the amount of overproduced protein (as a percent of total cellular protein) and the rate of LOH. Pearson’s correlation coefficient was not statistically significant: *r* = 0.160, *df* = 33, *p* = 0.718. Four of the 39 genes shown in Fig. 2—*FIN1, MOB2, OSH3*, and *SLM1*—were not included in this analysis, because the corresponding estimates of protein content were lost accidentally

The host strain, Y258, lacks the *PEP4* gene coding for proteinase A and, therefore, tends to accumulate peptides and proteins, including those with abnormal structures (Parr et al. 2007). We hypothesized that a very high overexpression of a single protein, irrespective of its function, could be a large enough burden for the cell to disturb mitosis. Figure 5 shows that this was not the case. Although the cellular content of proteins varied widely, there was no correlation between the amount of an expressed protein and the rate of LOH.

## Discussion

Stable replication and segregation of chromosomes requires concerted work of hundreds of proteins. It is difficult to predict a priori which of them are critically important and, even more so, which are not important for the stability of chromosomes. We used a collection of yeast diploid strains in which each single gene was abundantly overexpressed from a plasmid. The strains were heterozygous at the *CAN1* locus of chromosome V. This simple, although not yet tried experimental setting, proved successful as we found 39 genes that increased LOH when overexpressed. Of those, 22 are new for the CIN research (Supplementary Table S3).

Among the 39 genes increasing LOH upon overexpression, as many as 28 were within GO categories obviously linked to cell polarity and progression of cell cycle. Of those, as many as 13 related to sister chromatid segregation. There were also genes known to function primarily in meiosis and, therefore, possibly interfering with mitosis when expressed untimely. On top of the 28 genes directly linked to the cell cycle, there were four genes—*KRE8, MSN1, PCL8*, and *UGP1*—known as primarily involved in glucan metabolism. *β*-glucan is an essential component of the yeast cell wall, making more than half of its dry weight (Lesage and Bussey 2006). It is also a major component of the septum which forms between the mother and daughter cells (Roncero and Sánchez 2010). Disturbed metabolism of glucan results in improper development of a bud and septum which arrests the cell cycle prior to spindle body separation (Suzuki et al. 2004). This phenomenon has been called the cell wall integrity checkpoint (Negishi and Ohya 2010). Adding these four genes, as many as 32 out of the total of 39 genes increasing LOH can be related to cell cycle progression. This is hardly surprising. However, the paucity of genes directly involved in DNA metabolism is remarkable. Only one of the 39 gene products, the Top3 topoisomerase, is an enzyme for which DNA is a substrate. In former screens, much larger fraction of genes promoting LOH was linked to synthesis, recombination, transcription, or repair of DNA. This was especially true for gene deletions (Andersen et al. 2008; Eden et al. 2009a; Mikolaskova et al. 2018; Stirling et al. 2011). Our results suggest that overproduction of a single protein can be damaging to the coordination of complex processes necessary to complete cell division. In the case of DNA synthesis and repair, it is an absence rather than overabundance of a single protein that strongly interferes with the process of timely replication and separation of DNA strands. However, it is worth remembering that we used a collection of fusion proteins under truly heavy overexpression. It could be that not the core proteins, but their added components were more disturbing to some functions (cell division) than to others (DNA repair). In addition, overexpression is typically pleiotropic and generally proteotoxic. Therefore, caution is needed when making functional generalizations about the observed CIN patterns.

The seven remaining genes participate in the metabolism of sulfur compounds (among them, *SNO3* is only indirectly linked to this activity, and its specific role is still unknown). However, individual roles of these genes are different, even contradictory. For example, *GSH1* and *GLR1* code for a synthetase and reductase of glutathione, respectively. Glutathione is a major scavenger of reactive oxygen species, ROS (Deponte 2013), but it can impact, often indirectly, a variety of other cellular processes, including nuclear division (Fidai et al. 2016; Kaniak-Golik and Skoneczna 2015; Kumar et al. 2011; Matsuo et al. 2017). Deciphering the exact roles of these genes in maintaining genome stability would require work much broader than presented in this paper. We note, however, that the very strong destabilizing effect of Lsc2 observed here may have a straightforward explanation. Overabundance of this protein should result in an excessive level of succinate released from succinylo-CoA (Fleck and Brock 2009; Przybyla-Zawislak et al. 1998). Succinate is a source of electrons for the Complex II of the electron transport chain. Electrons escaping an excessively active transport chain form ROS which can be severely damaging to macromolecules, including DNA (Herrero et al. 2008).

Another result of our study is the observation that the proportion of whole-chromosome loss can vary substantially depending on medium. The strain used here hosted multiple copies of a 2-micron plasmid with a single yeast ORF fused to the *P*_*GAL1*_ promoter. One can safely assume that the number of plasmid copies per cell was high in glucose and raffinose and decreased after overexpression was turned on by galactose. This is, because the overexpressed protein is a burden, and therefore, cells with low numbers of plasmids are selected for (Makanae et al. 2013). Thus, not the very presence of abundant plasmid DNA but rather an intense transcription of its DNA was responsible for the observed high frequency of chromosome loss. Frequent unwinding and separation of DNA strands at the plasmid might have led to more intense interactions with chromosomes resulting in improper segregation of the latter. Indeed, it has been found that the 2-micron plasmid and chromosomes interact with each other. Specifically, the plasmid codes for a plasmid–chromosome recombination system which couples segregating chromosomes with plasmids to prevent both extinction and runaway proliferation of the latter (Mehta et al. 2002; Rizvi et al. 2017; Yen-Ting-Liu et al. 2014).

Several recent reviews attempted to list yeast genes which potentially affect the stability of chromosomes when their expression is absent or set at a non-native level (Choy et al. 2013; Duffy et al. 2016; Eden et al. 2009a; Stirling et al. 2011; Zhu et al. 2015). There are hundreds of such genes and the already long lists may not yet be complete. The stability of chromosomes is obviously a truly polygenic trait. Future research should probably concentrate on understanding which genes are important universally and which become relevant only under specific conditions.

## Electronic supplementary material

Below is the link to the electronic supplementary material.

Supplementary material 1 (XLSX 413 KB)

Supplementary material 2 (XLSX 17 KB)

Supplementary material 3 (XLSX 33 KB)
